# Comparison of the Declared Nutrient Content of Plant-Based Meat Substitutes and Corresponding Meat Products and Sausages in Germany

**DOI:** 10.3390/nu15183864

**Published:** 2023-09-05

**Authors:** Corinna Gréa, Anna Dittmann, David Wolff, Romy Werner, Christin Turban, Silvia Roser, Ingrid Hoffmann, Stefan Storcksdieck genannt Bonsmann

**Affiliations:** 1 Department of Nutritional Behaviour, Max Rubner-Institut (MRI)—Federal Research Institute of Food and Nutrition, Haid-und-Neu-Straße 9, 76131 Karlsruhe, Germany; corinna.grea@mri.bund.de (C.G.);; 2 Presidential Office, Max Rubner-Institut (MRI)—Federal Research Institute of Food and Nutrition, Haid-und-Neu-Straße 9, 76131 Karlsruhe, Germany

**Keywords:** plant-based meat substitute, meat products, sausages, vegetarian, alternative protein, reformulation

## Abstract

Plant-based meat substitutes (PBMS) are becoming increasingly popular due to growing concerns about health, animal welfare, and environmental issues associated with animal-based foods. The aim of this study was to compare the declared energy and nutrient contents of PBMS with corresponding meat products and sausages available on the German market. Mandatory nutrition labelling data of 424 PBMS and 1026 meat products and sausages, surveyed in 2021 and 2020, respectively, as part of the German national monitoring of packaged food were used to test for differences in energy and nutrient contents. Principal component analysis (PCA) was used to describe characteristics in the energy and nutrient contents. The comparison showed that most of the PBMS subcategories had significantly lower contents of fat and saturated fat but higher contents of carbohydrate and sugar than corresponding meat subcategories. For salt, the only striking difference was that PBMS salamis had lower salt content than meat salamis. Overall, the PCA revealed protein as a main characteristic for most PBMS categories, with the protein content being equivalent to or, in most protein-based PBMS, even higher than in the corresponding meat products. The wide nutrient content ranges within subcategories, especially for salt, reveal the need and potential for reformulation.

## 1. Introduction

It has long been known that a diet rich in, e.g., fruit and vegetables and low in red and processed meat is conducive to health. On the other hand, a high intake of red and processed meats and a low intake of fruits and vegetables are major dietary risk factors. According to the summaries of the health metrics on the global burden of diseases [1], diets low in fruit are responsible for 27.7 million DALYs (disease-adjusted life years), diets low in vegetables for 13.0 million DALYs, diets high in red meat for 23.9 million DALYs, and diets high in processed meat for 8.56 million DALYs. In addition, there is convincing evidence that a diet rich in highly processed foods supports weight gain [2], thus elevating the risk for conditions associated with excess body weight such as cardiovascular disease, diabetes, or cancer [3]. In recent years, and owing to the climate change debate, the health perspective concerning diet has been expanded to also consider planetary health [4,5,6]. Consequently, the environmental impact of diets has received increasing attention and has led to the concept of the planetary health diet [4]. Given the substantial environmental burden of animal-based foods, first and foremost meat and dairy from ruminants, a major tenet of environment-conscious dietary recommendations is to reduce meat and dairy consumption [4,5,6,7]. In response to this, a new market of plant-based meat substitutes (PBMS) has emerged.

Whether PBMS are suited to promote both human and environmental health is a matter of current debate [8,9]. Though the jury is out, the population’s growing interest in limiting meat consumption by substituting meat in their diet with PBMS has contributed to a growing demand for such products [10]. On the supply side, this is accompanied by rising sales of PBMS, a significant increase in product innovations, and a wide range of PBMS on the market [11]. In Germany, the production volume of PBMS increased from 60,400 tons in 2019 to 104,300 tons in 2022 (+72.7%) [12,13], with further increases predicted for the coming years [14]. The market offer comprises a large variety of vegan and vegetarian products that imitate the appearance, texture, and taste of meat products such as burger patties and sausages. The majority of these PBMS are based on grains, legumes, or protein-rich preparations thereof. Others may contain mycoproteins or ingredients of animal origin such as egg or milk [10,15].

The increasing popularity of PBMS gives cause to investigate their energy and nutrient contents in comparison to packaged meat products. Previous studies, mostly from outside Germany, assessed limited sets of PBMS products [16,17,18,19,20]. Consequently, a comprehensive assessment of PBMS products on the German market is lacking. Such an assessment is particularly important when consumers switch from meat products to PBMS with the intention of reducing their intake of saturated fat or salt. Recent studies showed that such health aspects may also influence consumer choices, although taste, appearance, or animal welfare are more important in this regard [21,22,23,24].

The present paper aims (1) to compare the declared energy and nutrient contents of PBMS to packaged meat products and sausages available in German retail and (2) to investigate characteristic properties in their nutritional composition.

## 2. Materials and Methods

### 2.1. Data Source and Management

As part of the German “National Reduction and Innovation Strategy (NRI) for Sugar, Fats, and Salt in processed foods” [25], the Federal Research Institute of Nutrition and Food (Max Rubner-Institut) has been mandated by the Federal Ministry of Food and Agriculture to monitor the energy and nutrient contents of selected packaged food categories between 2019 and 2025 [25,26]. For the present study, monitoring data such as energy and nutrient contents as declared in mandatory nutrition labelling (amount of fat, saturated fat, carbohydrate, sugars, protein, and salt), ingredient lists, and visuals of package fronts were used. Meat products and sausages were surveyed in 2020 [27], and PBMS in 2021 [28]. Corresponding product information was predominantly collected online by searching manufacturers’ websites manually within the defined survey period from August to December 2020 and 2021, respectively. In order to fill data gaps, the online research was complemented by enquiries with manufacturers and visits to grocery stores. The data were managed with a customised branded food module within the FoodCASE software, version 7.9.1 (Premotec GmbH, Winterthur, Switzerland). Detailed information on the design and methods of the German monitoring of packaged food is described elsewhere [29].

#### Inclusion Criteria and Definition of Food Categories

PBMS were included if the product name, the labelling, or the product description contained terms of an originally meat-based product such as schnitzel, (ham)burger, or sausage. PBMS without such textual reference to meat were only included if the design of the packaging or the product alluded to the original meat product. Products not aiming to imitate meat products, such as tofu blocks and dry products (e.g., granulated soy for reconstitution), were not considered.

For meat products and sausages, the large variety of products required focusing data collection on the top-selling product groups. Subcategories of meat products were differentiated according to the German guideline for meat and meat products [30] and the expertise of the MRI-department of Safety and Quality of Meat. For example, salami products are differentiated according to the degree of drying. For this study, we included only a selection of meat subcategories for which we monitored comparable subcategories of PBMS.

In order to compare the declared energy and nutrient contents of PBMS with those of corresponding meat products, each product was assigned to one of the following four categories: breaded products, minced products, sausages, and salamis. Within these, PBMS were further differentiated into protein-based and grain-/vegetable-based (hereafter: vegetable-based) PBMS (see Table 1). This distinction was mainly based on the information on the product. In case of declarations such as “based on plant protein” or if products were mainly made from tofu, milk, pulses, or nuts, they were categorised as protein-based PBMS. Products declared as vegetable products (e.g., vegetable nuggets) or mainly made from grains (e.g., oat flakes, rice), vegetables, or mushrooms were categorised as vegetable-based PBMS. If the information on the product and/or description did not provide the necessary information, the ingredient list was analysed to identify the basis or main ingredients of the product and thus allow proper categorisation. PBMS categorised as protein-based could contain sizable amounts of grains or vegetables, and vice versa. Owing to the small number of vegetarian products, no further distinction was made between vegetarian and vegan products.

In each of the four categories, meat products were further differentiated according to the main animal source, which could be pork, poultry, or beef. A summary of the categories used, their descriptions, the subcategories, and examples thereof can be found in Table 1. As the sample of vegetable-based sausages was too small for a comparison (*n* < 5), and no vegetable-based salamis were found, these two subcategories were not considered further.

### 2.2. Statistical Data Analysis

To compare the energy and nutrient contents of PBMS and meat, descriptive statistics including minimum, maximum, median, mean with standard deviation (SD), and quartiles (P25 and P75) were calculated for fat, saturated fat, carbohydrates, sugar, protein, salt (all in g/100 g), and energy content (in kcal/100 g) for each category and subcategory.

The normality of data distribution was checked using the Shapiro–Wilk test and rejected. To explore differences in energy and nutrient contents, the Kruskal–Wallis test was used within each category. Thereafter, each PBMS subcategory was compared against each of the meat subcategories by the Mann–Whitney non-parametric test for two independent samples with Bonferroni correction, resulting in a different number of comparisons across categories. For all results, a *p*-value below 0.05 was defined as statistically significant.

To discover potential clusters and describe characteristics in the energy and nutrient contents of PBMS and meat products within the four categories, principal component analysis (PCA) was applied in a further step. For this purpose, PBMS subcategories and meat subcategories were summarised, and a PCA was performed with four PBMS and four meat categories. All calculations of descriptive statistics and significance tests as well as all visualisations were conducted in R, version 4.2.2, and RStudio, version 2022.12.0+353 [30]. PCA was performed using the R package factoextra with scaled data [31].

## 3. Results

A total of 1450 products met the inclusion criteria, comprising 1026 meat products and sausages and 424 PBMS. The energy and nutrient contents (amount of fat, saturated fat, carbohydrates, sugars, protein, and salt) of these products were compared for the four categories of breaded products, minced products, sausages, and salamis, including testing of significant differences of PBMS subcategories to corresponding meat subcategories. For all four categories, significant differences were observed (*p* < 0.001). The observed differences of the pairwise comparisons are shown in Table 2.

### 3.1. Statistical Comparison of Energy and Nutrient Contents of PBMS and Meat Subcategories

#### 3.1.1. Breaded Products

Both breaded PBMS subcategories had significantly lower protein contents than the breaded meat subcategories. Furthermore, breaded protein-based PBMS showed significantly higher mean energy and carbohydrate contents, and breaded vegetable-based PBMS had higher carbohydrate and sugar contents than both breaded meat subcategories.

As for mean salt content, though breaded vegetable-based PBMS marked the lower boundary at 1.2 g/100 g, and breaded protein-based PBMS marked the upper boundary at 1.5 g/100 g, differences between PBMS and meat subcategories were not significant (Table 2).

#### 3.1.2. Minced Products

Minced PBMS subcategories had significantly lower contents of energy, fat, and saturated fat and higher contents of carbohydrates and sugar compared to individual or even all minced meat subcategories.

Minced protein-based PBMS had a significantly higher protein content than pork and a higher salt content than beef products. Differences from minced poultry products were only found for sugar content, which was significantly higher in minced protein-based PBMS.

Vegetable-based PBMS had significantly higher carbohydrate and sugar contents but a lower content of saturated fat compared to all minced meat categories and of salt compared to minced pork and beef products (Table 2).

#### 3.1.3. Sausages

Protein-based sausage substitutes differed from pork sausages in energy and all nutrients and from poultry sausages in all nutrients. They had lower fat, saturated fat, and salt contents than meat sausages and higher contents of carbohydrates, sugar, and protein (Table 2).

#### 3.1.4. Salamis

Protein-based salami substitutes differed from meat salamis in energy and all surveyed nutrients. They had lower mean contents in energy, fat, saturated fat, and salt than meat salami products, but they were higher in mean carbohydrate, sugar, and protein content (Table 2).

### 3.2. Characteristics in the Energy and Nutrient Contents of PBMS and Meat Products

**Figure 1:**
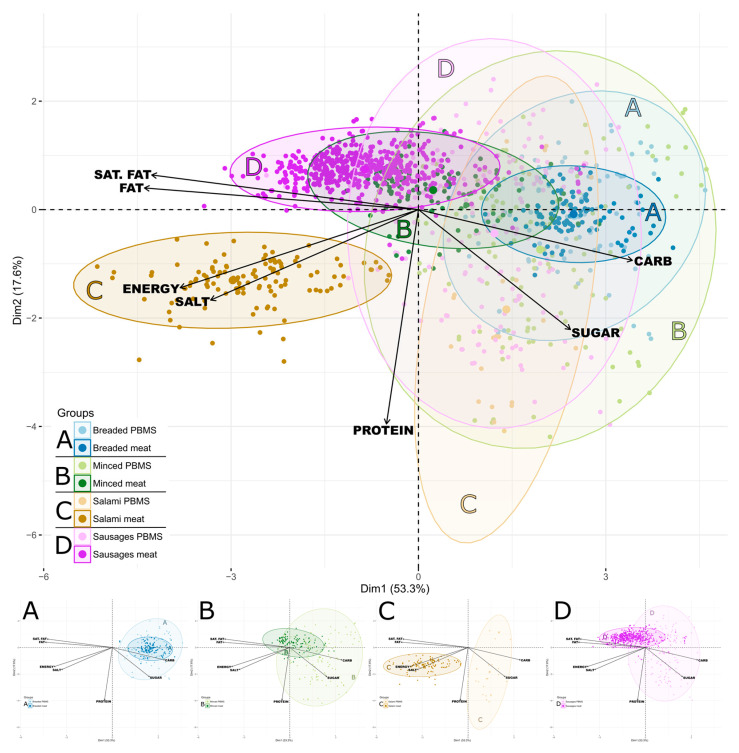
Principal component analysis (PCA) biplot of the PBMS and corresponding meat products and sausages grouped among eight categories. Each point represents the considered energy and nutrient contents of one product. Arrows indicate the contribution of the energy and nutrients to the difference; sat. fat = saturated fat; carb = carbohydrate. Each ellipse represents a 95% confidence interval under normality assumption of the PCA scores. Small figures part A–D illustrate the categories extracted from the overall PCA.

To investigate whether there were any characteristic attributes in the nutrient content of PBMS compared to meat, a PCA was carried out. The PCA provides information about interdependencies of the energy and nutrient contents of all examined products, grouped into the eight categories (Figure 1).

Here, the first two principal components explained 70.9% of the total variation in the product composition in the eight different categories. Except for protein, all energy and nutrient contents were distributed along the *x*-axis to some extent (Dim 1; 53.3%) and thus contributed to the first principal component. All PBMS categories showed relatively big clusters and wide distributions along the y-axis (Dim 2; 17.6%), where only the protein content seemed to be responsible for this distribution.

Except for salami (Figure 1, part C), all categories of meat products overlapped with their PBMS counterparts to a certain degree. These overlapping clusters indicate that the PBMS categories have a relatively similar composition compared to their corresponding meat categories at the level of energy and surveyed nutrients. This is especially apparent for breaded products.

**Figure A1:**
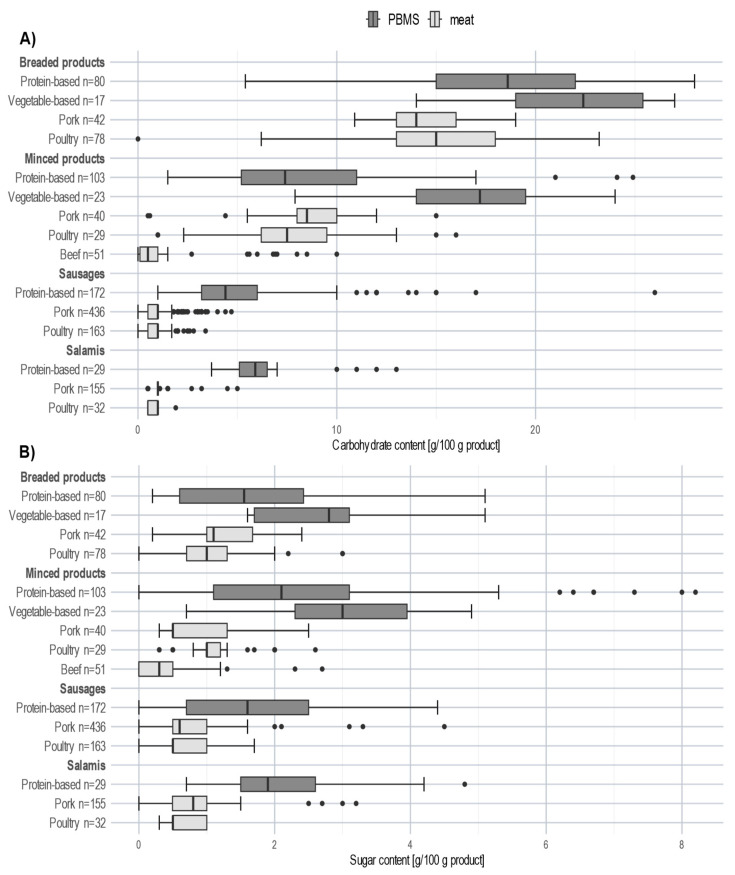
Distribution of carbohydrate (**A**) and sugar (**B**) contents of PBMS and meat subcategories; boxplot; ● represents an extreme value.

Clusters of PBMS categories were mainly located in the right half of the plot, showing that they are mainly described by their carbohydrate and sugar content (Figure 1). High carbohydrate and sugar contents of PBMS were already described before (Section 3.1): all PBMS subcategories except for one had significantly higher means and higher spans in carbohydrate and sugar contents than comparable meat products (Table 2; Figure A1). Breaded meat products represent an exception, as they are also mainly described through their carbohydrate content (Figure 1, part A).

**Figure 2:**
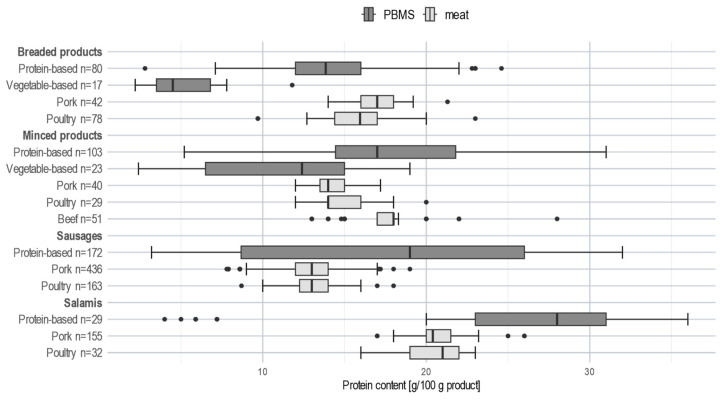
Distribution of protein content of PBMS and meat subcategories; boxplot; ● represents an extreme value.

Furthermore, minced PBMS, sausage substitutes, and salami substitutes were characterised by their protein content (Figure 1). The protein content of these PBMS categories showed a high variation compared to meat products (Table 2), as is illustrated in the PCA by the wide distribution along the *y*-axis. Overall, protein content was higher in these PBMS than in corresponding meat products. Almost 75% of the salami substitutes and 50% of the sausage substitutes exceeded the maximum protein content observed in the corresponding meat categories (Figure 2).

**Figure A2:**
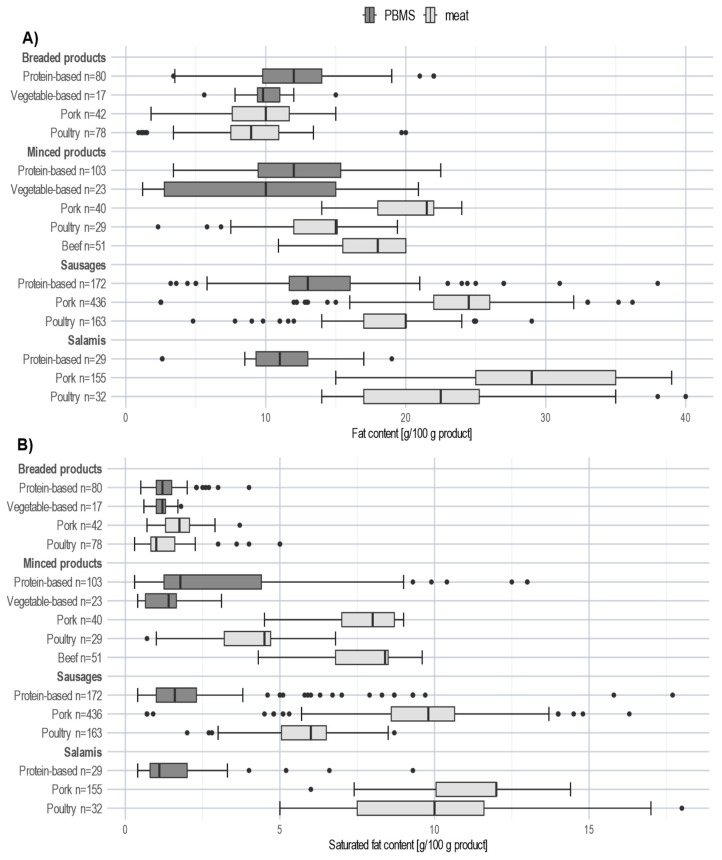
Distribution of fat (**A**) and saturated fat (**B**) contents of PBMS and meat subcategories; boxplot; ● represents an extreme value.

In contrast, three of the four meat clusters (salami, sausages, and half of the minced meat) are mainly located in the left half of the plot, which indicates that they are higher in salt, energy, fat, and saturated fat than their PBMS counterparts (Figure 1). The corresponding PBMS categories showed considerably lower medians for fat and saturated fat (Table 2; Figure A2).

Meat sausages were primarily characterised by their fat and saturated fat contents, and meat salami products are characterised by a particularly high salt and energy content. The cluster of minced meat products is altogether more centered, meaning that overall, no nutrient is dominant (Figure 1).

**Figure 3:**
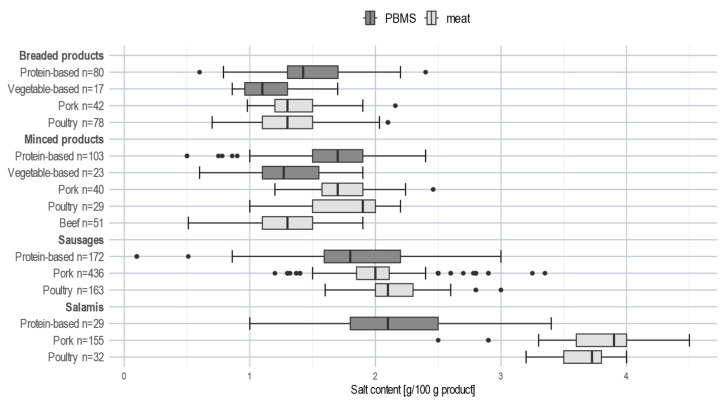
Distribution of salt content of PBMS and meat subcategories; boxplot; ● represents an extreme value.

PBMS and meat subcategories showed several significant differences in salt contents (Table 2). However, striking differences were only observed for salamis. According to the PCA, salt was only a characterising component for meat salamis (Figure 1). More than 75% of meat salamis exceeded a salt content of 3.5 g per 100 g product, whereas 75% of PBMS contained less than 2.5 g salt per 100 g (Figure 3).

## 4. Discussion

Using mandatory nutrition labelling data, the present analysis reports significant differences in energy and nutrient contents between PBMS and corresponding meat products and highlights characteristic properties in their nutritional composition.

When comparing the nutritional value of PBMS and meat products, protein as a valuable nutrient of meat and salt content as a health-related risk factor are often discussed [16,18,20,32,33,34,35,36,37].

For protein, several studies reported lower contents in PBMS than in meat products [16,19,20,32,33,34,35,36], except in some categories (especially sausages), where higher contents were observed [17,32,35,36]. In contrast, our PCA results indicated that protein is a main characteristic for most of the PBMS categories. This may partly be explained by the fact that more protein-based than vegetable-based products were included in the analysis. Compared to the corresponding meat products, the detailed sub-categorisation showed lower protein contents for vegetable-based and, except for breaded products, equivalent or higher protein contents for protein-based PBMS (Table 2; Figure 2). Apart from the different product categorisation, these discrepancies may also be due to country-specific differences in terms of market offer and product formulation. The differentiation between protein-based and vegetable-based PBMS might also be important for consumers looking to replace meat as a protein source with a protein-rich plant-based option. In this context, it is worth noting that the monitored PBMS were often found to bear a protein-related nutrition claim according to the EU Regulation [38].

The second important nutrient in the evaluation of PBMS is salt, as PBMS have been reported to contain significant amounts of salt, and especially to be higher in salt compared to meat products [16,19,20,33,35,37,39]. Only for plant-based sausage substitutes, some studies reported lower salt contents compared to meat sausages [16,17,18,32,33,35,36,37]. Our analysis showed lower mean salt contents for most PBMS subcategories relative to meat, although the differences in absolute content were mostly only small (except for salamis). The mean salt content of both meat salami subcategories is 1.8 times higher than the mean salt content of protein-based salami substitutes. The wide range and, in some cases, high content of salt found in meat products and PBMS reveal the potential and need for reformulation.

In Germany, salt reduction commitments for meat products within the NRI are limited to reducing particularly high salt contents (referred to as salt peaks) in cooked meat products [25]. In the UK, specific salt reduction targets have been set for PBMS such as sausages, burgers, or flavoured meat substitutes (combined under the generic term meat-free products (on average, ≤0.85 g salt/100 g meat-free product)) as well as for meat-free bacon (≤1.78 g salt/100 g) and plain meat alternatives (≤0.63 g salt/100 g) [40]. These targets are only met by a subset of PBMS on the UK market (36% (meat-free products) and 15% (meat-free bacon), respectively) [16]. In the present study, only a few products comply with those targets.

According to our results, PBMS on the German market are mainly characterised by their carbohydrate and sugar contents, whereas meat products showed a higher loading in the PCA for fat and saturated fat, or for salt and energy. Though there may well be a link between total and saturated fat content, the observed pairing of salt and energy is less obvious (see Figure 1).

Due to the use of various starchy ingredients, a higher carbohydrate and sugar content is to be expected in PBMS, and this has already been shown in previous studies [17,18,19,32,34,35,36,37,39]. Smaller differences in sugar content between breaded and minced protein-based PBMS and the respective meat products can be explained by similar ingredients such as breadcrumbs, bread, or cornflakes used for the breading. Even though the sugar content was higher in PBMS than in meat products, it did not exceed 5 g/100 g product, which is the maximum amount allowed by the EU nutrition and health claims regulation [38] for classifying a product as “low in sugar”. Thus, our results are in line with an earlier, smaller market analysis in Germany [41], which showed that many PBMS could be considered as “low in sugar” according to the regulation. In addition, PBMS have been reported to have high levels of dietary fibre [16,18,19,20,32,33] or to qualify as a source of dietary fibre [36], as many of the main ingredients used are naturally high in dietary fibre. However, a direct comparison of dietary fibre was not part of this study, as this is not a component of the mandatory nutrition labelling in the EU.

Meat products, especially sausages, are predominantly described by their contents of fat and saturated fat, which is to be expected due to the traditional production with different animal fat components [42]. On the other hand, PBMS often show a wide range of fat and saturated fat contents [16,20,33,34,36,39,43], an observation supported by the present analysis. Although the ingredient lists were not examined in detail here, this finding is likely attributable to the different fat sources used, e.g., rapeseed oil compared to coconut or palm oil [34,41,44,45]. This variability within the groups indicates that it is possible to manufacture PBMS with low saturated fat content without compromising the product properties. The range also allows consumers to select products with lower total or saturated fat contents.

Which PBMS products people choose, the quantity consumed, the amount of meat replaced, and any wider dietary changes resulting from using PBMS all influence nutrient intake and nutritional status, which in turn impact health. Just looking at the nutritional contribution of PBMS, our results indicate that, with the exception of the category of breaded products, a majority of PBMS may be suited to reduce total and saturated fat intake. Regarding salt, lower intakes may be achieved by switching from meat salamis to the corresponding protein-based PBMS. In the breaded and minced product categories, the same holds for switching to vegetable-based PBMS. Therefore, PBMS may also help reduce the disease burden associated with diets high in sodium [1]. Among the investigated PBMS, there were products that combine lower levels of total fat, saturated fat, and sodium. However, it would be wrong to assume that simply switching from meat to the corresponding substitute category would result in lower intakes of said nutrients by default.

A potential downside of consuming PBMS lies in the fact that these products are often highly processed, not least because of isolated plant derivatives being used for their production [9]. Such processing may result in noticeably lower contents of bioactive compounds that are abundant in whole plant foods [8,9] while producing foods that are quick to consume. Diets high in processed, quick-to-eat foods have been shown to promote weight gain [2] and so represent a dietary risk factor for associated morbidities. As another limitation, PBMS may fail to provide essential micronutrients such as vitamin B_12_ or highly bioavailable iron as found in meat.

On the whole, if PBMS are used to replace meat in the diet, this is likely to reduce the health risks associated with high meat consumption [1] as well as the environmental impact of the diet [6,7,9]. Nonetheless, evidence from food compositional studies and epidemiologic investigations is needed, as outlined below, to determine whether PBMS can support population health in the long term.

### 4.1. Strength and Limitations

One strength of the present study is its comprehensive data set, with product information gathered systematically via online research on manufacturer and major retail websites and visits to retail stores. The data set comprises food products of brand manufactures and discount brands. However, Germany has a vast range of meat products and sausages, which are furthermore characterised by numerous regional specialities. For this reason, the monitoring of this product category had to be limited to a manageable number of subcategories [27]. At the same time, the market for PBMS is growing rapidly and thus challenging to capture. Nonetheless, the present study is based on systematically collected nutritional data for a broad range of PBMS and meat products and, within the analysed product subcategories, can be seen as covering the German market.

As a result of the observed variety of PBMS on the German market, we differentiated between protein-based and vegetable-based products wherever possible. On the one hand, this allows for considering existing in-group variabilities among more consistent PBMS subgroups in the analysis. This is important, as protein-based PBMS in particular might be intended to mimic not only the appearance but also the nutrient profile of corresponding meat products, whereas vegetable-based products do not. On the other hand, the more detailed categorisation results in smaller subgroups and limits the comparability to other studies. It is worth noting that this distinction was not feasible for the breaded and minced categories in the PCA due to the small sample sizes in the vegetable-based subcategories.

The present comparison of energy and nutrient contents between PBMS and meat products is based on mandatory nutrition labelling on packaged products. These values are either based on the chemical analysis of the whole food, calculations based on the known or actual average values of the ingredients used, or a calculation based on average values published in nutrient databases. It is fair to assume that the gathered values represent a mixture of these approaches. Thus, it is possible that our results differ from the results of others where the nutritional values were determined using only one of these approaches. However, this applies to all pre-packaged foods, not just to PBMS or meat products. In this context, it should be noted that the EU regulations concerning mandatory nutrition labelling include a tolerated discrepancy of up to ±20% between the declared values and what would be found in a chemical analysis of the food [46].

A further limitation lies in the fact that the declared nutrient content is insufficient to make a full assessment of the nutritional equivalence (or absence thereof) of PBMS and their meat counterparts. Considering the rather high intake of salt and saturated fat in Germany [47,48], our data suggest that some PBMS currently on the market can serve as an alternative with nutritional benefits in comparison to meat, but the choice seems more limited for salt than for saturated fat.

### 4.2. Future Research

This study focused on the evaluation of declared energy and nutrient contents surveyed in 2021 (PBMS) and 2020 (meat) within the German national product monitoring. As the monitoring continues, data from future surveys will allow for comparing the contents and changes in energy and nutrients of PBMS and meat products over several points in time. With these data alone, an overall assessment of the nutritional and health value of PBMS in comparison to meat products is not possible. On the basis of product labelling information, a first further step would be to complement the data by an evaluation of a summarising score, for example, the Nutri-Score. The Nutri-Score is the officially adopted front-of-pack nutrition labelling system in Germany [49]. However, declaration of the Nutri-Score on food packaging is voluntary (according to Regulation (EU) No. 1169/2011 [50]) and is only used by some producers and retailers in Germany. Thus, a comparison of the distribution of the Nutri-Score between the different product subcategories as performed in other studies [17,18,20,35] requires its calculation based on the ingredient lists. A major challenge here remains that the mandatory food labelling does not provide all the information needed to calculate the Nutri-Score, therefore requiring certain assumptions with the inherent risk for error. Another approach could be an in-depth analysis of the ingredient lists, including food additives, with a view to gauging the degree of processing. A review of the main ingredients or protein/fat sources used would already allow for an initial assessment of the protein and fat quality, which are important aspects in the context of comparisons with meat products.

To better understand the extent to which PBMS on the German market are nutritionally equivalent to their corresponding meat products, analyses well beyond the nutrition labelling information are required. Such analyses might focus on protein quality (amino acid profile), occurrence and bioavailability of micronutrients (as done, e.g., by [35,43,44,45]), as well as possible contaminants in PBMS compared to meat. Considering the large number of possible ingredients in PBMS, future analyses may also include digestibility and allergenic potential. These chemical and biological analyses would help to gain a more comprehensive understanding of the nutritional differences of PBMS and corresponding meat products. However, these analyses are cost- and time-intensive and therefore would not be feasible to perform for the entire set of products considered in this study.

Beyond nutritional evaluations, future analyses regarding environmental impacts should consider the effects on biodiversity and land use, which have been given little consideration so far, when comparing PBMS to their meat counterparts. Though processed foods in general have been associated with a potentially high impact [10,51,52,53], at times, PBMS are being marketed as more environmentally friendly than corresponding meat products [8]. A differentiated consideration of products is important, especially with regard to the degree of processing/processing method of the ingredients as well as their origin and cultivation/production conditions, as, e.g., the environmental impact varies depending on these qualities, as already shown for PBMS and meat burgers [54]. The detailed differentiation into vegetable- and protein-based products used as well as the overall subdivision in product types (e.g., breaded vegetable-based PBMS vs. breaded poultry products) could be a first approach to describe, e.g., the environmental impacts of PBMS compared to meat with the warranted degree of resolution.

## 5. Conclusions

Using mandatory nutrition labelling data, this study is the first to compare the energy and nutrient contents for such a comprehensive data set of PBMS and corresponding meat products and sausages on the German market.

At the level of the declared nutrients, among the four categories, breaded products of PBMS and meat were most similar in overall composition, and salamis were most different.

At the single nutrient level, similarities between PBMS and meat were found for protein in protein-based minced PBMS, and higher protein contents were found in sausage and salami substitutes. Major differences were related to saturated fat content (lower especially in PBMS versions of minced, sausage, and salami products) and salt content (especially lower in salami substitutes). From a consumer perspective, lowering one’s salt intake could be an important reason for switching from meat to PBMS. However, our results show that such a switch requires checking the label to ensure the substitute does have a lower salt content. On the whole, PBMS cannot be classified as generally superior or inferior to meat products solely considering the declared nutrient content.

The wide ranges in nutrient content in the surveyed subcategories indicate the potential for reformulation, particularly for salt and saturated fat. At the same time, the observed product variety already offers consumers the opportunity to choose nutritionally more favourable products within and between PBMS subcategories.

These data represent the first step towards a health evaluation of PBMS on the German market. For a more comprehensive assessment of PBMS in comparison to meat products, further analyses of the overall composition and bioavailability of macro- and micronutrients and an evaluation of the role of these products in the overall diet, including frequency of consumption and variety of the diet in general, are required.

## Figures and Tables

**Table 1:** Food categories and subcategories of plant-based meat substitutes (PBMS) as well as the corresponding meat products and sausages.

Category	Description	Subcategory	Examples
Breaded products	Breaded or battered products, including breaded or battered burgers/patties and the corresponding PBMS; only products without filling	Protein-based PBMS	Chicken-style nuggets; veggie schnitzel; crispy chicken-style burger
		Vegetable-based PBMS	Vegetable schnitzel; rice nuggets; broccoli burger
		Pork	Pork schnitzel; schnitzel vienna style
		Poultry	Chicken or turkey schnitzel; chicken nuggets/sticks/chips; crispy chicken burger
Minced products	Products made from minced meat and the corresponding PBMS	Protein-based PBMS	Black bean burger; veggie balls
		Vegetable-based PBMS	Jackfruit burger, sweet potato patties
		Pork	Meatballs; cevapcici
		Poultry	Chicken meatballs; chicken cevapcici
		Beef	Beef burger; beef burger patties
Sausages	Typical German cooked sausages and the corresponding PBMS intended for cold or warm consumption; meat products may be cured; sausages as a whole or sliced	Protein-based PBMS ^1^	Seitan cold cuts; veggie cocktail sausages; soy sausage
		Pork	“Lyoner”, meat sausage, wiener sausage, bratwurst
		Poultry	Chicken sausage; poultry bratwurst
Salamis	Salami and PBMS for raw sausages; salami as a whole and sliced	Protein-based PBMS ^2^	Tofu salamito; vegan garlic salami
		Pork ^3^	Salami cold cuts; pepper salami
		Poultry ^4^	Turkey salami; chicken salami

^1^ The sample size of vegetable-based sausages was too small for a comparison; ^2^ no vegetable-based salamis were found; ^3^ salami produced with ≤130 g raw meat per 100 g salami, ^4^ salami produced with ≤120 g raw meat per 100 g salami; for detailed information on the definition of product subcategories, see [27].

**Table 2:** Energy and nutrient contents of PBMS and corresponding meat products and sausages per subcategory across four categories, based on mandatory nutrition labelling (per 100 g product).

		Breaded Products				Minced Products					Sausages			Salamis		
		PBMS ^1^		Meat		PBMS ^2^		Meat			PBMS ^3^	Meat		PBMS ^4^	Meat	
		Protein	Vegetable	Pork	Poultry	Protein	Vegetable	Pork	Poultry	Beef	Protein	Pork	Poultry	Protein	Pork	Poultry
	Count	80	17	42	78	103	23	40	29	51	172	436	163	29	155	32
**ENERGY** [kcal/100 g]	Mean ± SD	245 ± 41 ^a,b^	206 ± 30	210 ± 32	203 ± 37	225 ± 44 ^a^	207 ± 71 ^a^	275 ± 25	216 ± 36	236 ± 21	222 ± 46 ^a^	271 ± 40	228 ± 28	227 ± 41 ^a,b^	347 ± 43	290 ± 60
	Median	239	209	215	203	226	216	281	222	245	226	275	232	240	349	292
	Min/Max	141/339	131/248	137/272	129/302	118/341	122/330	206/328	112/272	180/270	127/395	88/370	105/314	135/275	220/431	216/432
**FAT** [g/100 g]	Mean ± SD	12.1 ± 4.0 ^b^	10.0 ± 2.1	9.3 ± 3.2	8.6 ± 3.8	12.6 ± 4.2 ^a,c^	9.5 ± 6.3 ^a,c^	20.2 ± 2.8	13.8 ± 4.2	17.6 ± 2.6	14.1 ± 4.9 ^a,b^	23.9 ± 4.6	18.9 ± 3.3	11.2 ± 4.1 ^a,b^	28.9 ± 4.9	22.8 ± 7.3
	Median	12.0	9.8	10.0	9.0	12.0	10.0	21.5	15.0	18.0	13.0	24.5	20.0	11.0	29.0	22.5
	Min/Max	3.4/22.0	5.6/15.0	1.8/15.0	0.9/20.0	3.4/22.5	1.2/20.9	14.0/24.0	2.3/19.4	10.9/20.0	3.2/38.0	2.5/36.2	4.8/29.0	2.6/19.0	15.0/39.0	14.0/40.0
**SAT. FAT** [g/100 g]	Mean ± SD	1.4 ± 0.6 ^a^	1.2 ± 0.4 ^a^	1.8 ± 0.7	1.3 ± 0.9	3.2 ± 3.0 ^a,c^	1.3 ± 0.7 ^a,b,c^	7.7 ± 1.2	4 ± 1.4	7.7 ± 1.4	2.3 ± 2.5 ^a,b^	9.5 ± 2	5.9 ± 1.3	2.0 ± 2.0 ^a,b^	11.2 ± 1.4	10.0 ± 3.1
	Median	1.2	1.2	1.8	1.0	1.8	1.4	8.0	4.5	8.4	1.6	9.8	6.0	1.1	12.0	10.0
	Min/Max	0.5/4.0	0.6/1.8	0.7/3.7	0.3/5.0	0.3/13.0	0.4/3.1	4.5/9.0	0.7/6.8	4.3/9.6	0.4/17.7	0.7/16.3	2.0/8.7	0.4/9.3	6.0/14.4	5.0/18.0
**CARB** [g/100 g]	Mean ± SD	18.3 ± 5.1 ^a,b^	22.0 ± 4.5 ^a,b^	14.4 ± 2.3	15.3 ± 3.9	8.3 ± 4.5 ^c^	16.7 ± 4.1 ^a,b,c^	8.5 ± 2.7	7.8 ± 3.7	1.7 ± 2.7	5.1 ± 3.2 ^a,b^	1.0 ± 0.7	1.0 ± 0.5	6.4 ± 2.3 ^a,b^	1.1 ± 0.5	0.9 ± 0.3
	Median	18.6	22.4	14.0	15.0	7.4	17.2	8.5	7.5	0.5	4.4	1.0	1.0	5.9	1.0	1.0
	Min/Max	5.4/28.0	14.0/27.0	10.9/19.0	0/23.2	1.5/24.9	7.9/24.0	0.5/15.0	1.0/16.0	0/10.0	1.0/26.0	0/4.7	0/3.4	3.7/13.0	0.5/5.0	0.5/1.9
**SUGAR** [g/100 g]	Mean ± SD	1.7 ± 1.2	2.6 ± 0.9 ^a,b^	1.3 ± 0.5	1.1 ± 0.6	2.5 ± 1.8 ^a,b,c^	3.0 ± 1.2 ^a,b,c^	0.9 ± 0.6	1.1 ± 0.5	0.4 ± 0.6	1.6 ± 1.0 ^a,b^	0.7 ± 0.4	0.7 ± 0.3	2.2 ± 1.0 ^a,b^	0.7 ± 0.6	0.7 ± 0.3
	Median	1.6	2.8	1.1	1.0	2.1	3.0	0.5	1.0	0.3	1.6	0.6	0.5	1.9	0.8	0.5
	Min/Max	0.2/5.1	1.6/5.1	0.2/2.4	0/3.0	0/8.2	0.7/4.9	0.3/2.5	0.3/2.6	0/2.7	0/4.4	0/4.5	0/1.7	0.7/4.8	0/3.2	0.3/1.0
**PROTEIN** [g/100 g]	Mean ± SD	13.9 ± 3.9 ^a,b^	5.3 ± 2.4 ^a,b^	17.0 ± 1.4	15.8 ± 2.2	18.0 ± 6.1 ^a^	11.1 ± 5.6 ^c^	14.4 ± 1.4	14.8 ± 1.7	17.6 ± 2.4	17.9 ± 8.8 ^a,b^	13.0 ± 1.6	13.4 ± 1.4	24.2 ± 10.4 ^a,b^	20.6 ± 1.4	20.3 ± 1.9
	Median	13.9	4.5	17.0	16.0	17.0	12.4	14.0	14.0	18.0	19.0	13.0	13.0	28.0	20.4	21.0
	Min/Max	2.8/24.6	2.2/11.8	14.0/21.3	9.7/23.0	5.2/31.0	2.4/19.0	12.0/17.2	12.0/20.0	13.0/28.0	3.2/32.0	7.8/19.0	8.7/18.0	4.0/36.0	17.0/26.0	16.0/23.0
**SALT** [g/100 g]	Mean ± SD	1.5 ± 0.4	1.2 ± 0.3	1.4 ± 0.3	1.3 ± 0.3	1.9 ± 0.5 ^c^	1.3 ± 0.3 ^a,b^	1.8 ± 0.3	1.8 ± 0.3	1.3 ± 0.3	1.9 ± 0.5 ^a,b^	2.0 ± 0.3	2.1 ± 0.2	2.1 ± 0.6 ^a,b^	3.8 ± 0.3	3.7 ± 0.2
	Median	1.4	1.1	1.3	1.3	1.7	1.3	1.7	1.9	1.3	1.8	2.0	2.1	2.1	3.9	3.7
	Min/Max	0.6/2.4	0.9/1.7	1.0/2.2	0.7/2.1	0.5/2.4	0.6/1.9	1.2/2.5	1.0/2.2	0.5/1.9	0.1/3	1.2/3.4	1.6/3.0	1.0/3.4	2.5/4.5	3.2/4.0

^a,b,c^ Superscript letters indicate a significant difference (*p* < 0.05) between the PBMS subcategory and the corresponding meat subcategory identified by the letter, based on the code: ^a^ = pork, ^b^ = poultry, ^c^ = beef; SAT. FAT = saturated fat; CARB = carbohydrate; ^1^ 80 vegan and 17 vegetarian breaded PBMS; ^2^ 113 vegan and 13 vegetarian minced PBMS; ^3^ 135 vegan and 37 vegetarian sausage PBMS; ^4^ 28 vegan and 1 vegetarian salami PBMS.

## Data Availability

Data are available, on reasonable request, from the corresponding author.

## References

[1] The Lancet Global Burden of Diseases. GBD Cause and Risk Summaries. https://www.thelancet.com/gbd/summaries.

[2] Hall K.D., Ayuketah A., Brychta R., Cai H., Cassimatis T., Chen K.Y., Chung S.T., Costa E., Courville A., Darcey V. (2019). Ultra-processed diets cause excess calorie intake and weight gain: An inpatient randomized controlled trial of ad libitum food intake. Cell Metab..

[3] Guh D.P., Zhang W., Bansback N., Amarsi Z., Birmingham C.L., Anis A.H. (2009). The incidence of co-morbidities related to obesity and overweight: A systematic review and meta-analysis. BMC Public Health.

[4] Willett W., Rockström J., Loken B., Springmann M., Lang T., Vermeulen S., Garnett T., Tilman D., DeClerck F., Wood A. (2019). Food in the Anthropocene: The EAT–Lancet Commission on healthy diets from sustainable food systems. Lancet.

[5] Shukla P.R., Skea J., Calvo Buendia E., Masson-Delmotte V., Pörtner H.-O., Roberts D.C., Zhai P., Slade R., Connors S., van Diemen R., IPCC (2019). Summary for Policymakers. Climate Change and Land: An IPCC Special Report on Climate Change, Desertification, Land Degradation, Sustainable Land Management, Food Security, and Greenhouse Gas Fluxes in Terrestrial Ecosystems.

[6] Stehfest E., Bouwman L., Van Vuuren D.P., Den Elzen M.G., Eickhout B., Kabat P. (2009). Climate benefits of changing diet. Clim. Chang..

[7] Scarborough P., Clark M., Cobiac L., Papier K., Knuppel A., Lynch J., Harrington R., Key T., Springmann M. (2023). Vegans, vegetarians, fish-eaters and meat-eaters in the UK show discrepant environmental impacts. Nat. Food.

[8] Van Vliet S., Kronberg S.L., Provenza F.D. (2020). Plant-based meats, human health, and climate change. Front. Sustain. Food Syst..

[9] Toh D.W.K., Srv A., Henry C.J. (2022). Unknown impacts of plant-based meat alternatives on long-term health. Nat. Food.

[10] Alcorta A., Porta A., Tárrega A., Dolores Alvarez M., Vaquero M.P. (2021). Foods for plant-based diets: Challenges and innovations. Foods.

[11] Mintel (2022). GNPD. https://www.mintel.com/de/produkte/gnpd/.

[12] Destatis Upward Trend for Meat Substitutes Continued: Production Increased by 17% in 2021 Year on Year. Press Release No. N 025 of 9 May 2022. https://www.destatis.de/EN/Press/2022/05/PE22_N025_42.html.

[13] Destatis Fleischersatz Weiter im Trend: Produktion Steigt um 6.5% Gegenüber 2021. Pressemitteilung Nr. N 027 vom 10. Mai 2023. https://www.destatis.de/DE/Presse/Pressemitteilungen/2023/05/PD23_N027_42.html.

[14] Fortune Business Insights. Meat Substitutes Market Size, Share and COVID-19 Impact Analysis. https://www.fortunebusinessinsights.com/industry-reports/meat-substitutes-market-100239.

[15] Kumar P., Chatli M., Mehta N., Singh P., Malav O., Verma A.K. (2017). Meat analogues: Health promising sustainable meat substitutes. Crit. Rev. Food Sci. Nutr..

[16] Alessandrini R., Brown M.K., Pombo-Rodrigues S., Bhageerutty S., He F.J., MacGregor G.A. (2021). Nutritional quality of plant-based meat products available in the UK: A cross-sectional survey. Nutrients.

[17] Katidi A., Xypolitaki K., Vlassopoulos A., Kapsokefalou M. (2023). Nutritional quality of plant-based meat and dairy imitation products and comparison with animal-based counterparts. Nutrients.

[18] Cutroneo S., Angelino D., Tedeschi T., Pellegrini N., Martini D. (2022). Nutritional quality of meat analogues: Results from the Food Labelling of Italian Products (FLIP) Project. Front. Nutr..

[19] Melville H., Shahid M., Gaines A., McKenzie B.L., Alessandrini R., Trieu K., Wu J.H., Rosewarne E., Coyle D.H. (2022). The nutritional profile of plant-based meat analogues available for sale in Australia. Nutr. Diet..

[20] Bryngelsson S., Moshtaghian H., Bianchi M., Hallström E. (2022). Nutritional assessment of plant-based meat analogues on the Swedish market. Int. J. Food Sci. Nutr..

[21] Weinrich R. (2019). Opportunities for the adoption of health-based sustainable dietary patterns: A review on consumer research of meat substitutes. Sustainability.

[22] He J., Evans N.M., Liu H., Shao S. (2020). A review of research on plant-based meat alternatives: Driving forces, history, manufacturing, and consumer attitudes. Compr. Rev. Food Sci. Food Saf..

[23] Hoek A.C., Luning P.A., Weijzen P., Engels W., Kok F.J., de Graaf C. (2011). Replacement of meat by meat substitutes. A survey on person- and product-related factors in consumer acceptance. Appetite.

[24] Michel F., Hartmann C., Siegrist M. (2021). Consumers’ associations, perceptions and acceptance of meat and plant-based meat alternatives. Food Qual. Prefer..

[25] Federal Ministry of Food and Agriculture The National Reduction and Innovation Strategy for Sugar, Fats and Salt in Processed Foods. https://www.bmel.de/EN/topics/food-and-nutrition/healthy-diet/reduction-innovation-strategy-less-sugar-fat-salt.html.

[26] Max Rubner-Institute Reducing the Salt, Sugar, and Fat Contents in Food. https://www.mri.bund.de/en/topics/reduktion-von-zucker-fett-und-salz/.

[27] Demuth I., Busl L., Ehnle-Lossos M., Elflein A., Fark N., Goos E., Turban C., Werner L., Werner R., Storcksdieck genannt Bonsmann S. (2021). Produktmonitoring 2020 Ergebnisbericht.

[28] Gréa C., Busl L., Dittmann A., Ehnle-Lossos M., Elflein A., Fark N., Goos E., Turban C., Werner R., Wolff D. (2022). Produktmonitoring 2021 Ergebnisbericht, Version 2.0.

[29] Gréa C., Turban C., Roser S., Storcksdieck genannt Bonsmann S., Hoffmann I. (2023). Design and methods of the German monitoring of packaged food in the European context. J. Food Compos. Anal..

[30] Deutsche Lebensmittelbuch Kommission Leitsätze für Fleisch und Fleischerzeugnisse Neufassung vom 25.11.2015 (BAnz AT 23.12.2015 B4, GMBl 2015 S. 1357), zuletzt geändert durch die Bekanntmachung vom 23.09.2020 (BAnz AT 29.10.2020 B4, GMBl 45/2020 S. 971). https://www.deutsche-lebensmittelbuch-kommission.de/leitsaetze.

[31] R Core Team (2013). R: A Language and Environment for Statistical Computing. http://www.R-project.org/.

[32] Kassambara A. (2016). Factoextra: Extract and visualize the results of multivariate data analyses. R Package Version.

[33] Tonheim L.E., Austad E., Torheim L.E., Henjum S. (2022). Plant-based meat and dairy substitutes on the Norwegian market: Comparing macronutrient content in substitutes with equivalent meat and dairy products. J. Nutr. Sci..

[34] Cole E., Goeler-Slough N., Cox A., Nolden A. (2022). Examination of the nutritional composition of alternative beef burgers available in the United States. Int. J. Food Sci. Nutr..

[35] Boukid F., Castellari M. (2021). Veggie burgers in the EU market: A nutritional challenge?. Eur. Food Res. Technol..

[36] Pointke M., Pawelzik E. (2022). Plant-based alternative products: Are they healthy alternatives? Micro-and macronutrients and nutritional scoring. Nutrients.

[37] Curtain F., Grafenauer S. (2019). Plant-based meat substitutes in the flexitarian age: An audit of products on supermarket shelves. Nutrients.

[38] Romão B., Botelho R.B.A., Nakano E.Y., Raposo A., Han H., Vega-Muñoz A., Ariza-Montes A., Zandonadi R.P. (2022). Are vegan alternatives to meat products healthy? A study on nutrients and main ingredients of products commercialized in Brazil. Front. Public Health.

[39] European Parliament, European Council Regulation (EC) No 1924/2006 at the European Parliament and of the Council of 20 December 2006 on Nutrition and Health Claims Made on Foods. https://eur-lex.europa.eu/legal-content/EN/TXT/HTML/?uri=CELEX:02006R1924-20141213&from=EN.

[40] Falkenberg C., Trexler A., Garaus C., Pöchtrager S. (2023). Meat substitute markets: A comparative analysis of meat analogs in Austria. Foods.

[41] Public Health England Salt Reduction Targets for 2024. https://assets.publishing.service.gov.uk/government/uploads/system/uploads/attachment_data/file/915406/2024_salt_reduction_targets_070920-FINAL-1.pdf.

[42] Huber J., Keller M. Ernährungsphysiologische Bewertung von Konventionell und Ökologisch Erzeugten Vegetarischen und Veganen Fleisch- und Wurstalternativen. Studie im Auftrag der Albert Schweitzer Stiftung für Unsere Mitwelt. www.albert-schweitzer-stiftung.de/fleischalternativen-studie.

[43] Harnack L., Mork S., Valluri S., Weber C., Schmitz K., Stevenson J., Pettit J. (2021). Nutrient composition of a selection of plant-based ground beef alternative products available in the United States. J. Acad. Nutr. Diet..

[44] De Marchi M., Costa A., Pozza M., Goi A., Manuelian C.L. (2021). Detailed characterization of plant-based burgers. Sci. Rep..

[45] Mayer Labba I.-C., Steinhausen H., Almius L., Bach Knudsen K.E., Sandberg A.-S. (2022). Nutritional composition and estimated iron and zinc bioavailability of meat substitutes available on the swedish market. Nutrients.

[46] European Comission Health, Consumers Directorate-General (2012). Guidance Document for Competent Authorities for the Control of Compliance with EU Legislation on: Regulation (EU) No 1169/2011 of the European Parliament and of the Council of 25 October 2011 on the Provision of Food Information to Consumers, Amending Regulations (EC) No 1924/2006 and (EC) No 1925/2006 of the European Parliament and of the Council, and Repealing Commission Directive 87/250/EEC, Council Directive 90/496/EEC, Commission Directive 1999/10/EC, Directive 2000/13/EC of the European Parliament and of the Council, Commission Directives 2002/67/EC and 2008/5/EC and Commission Regulation (EC) No 608/2004 and Council Directive 90/496/EEC of 24 September 1990 on Nutrition Labelling of Foodstuffs and Directive 2002/46/EC of the European Parliament and of the Council of 10 June 2002 on the Approximation of the Laws of the Member States Relating to Food Supplements with Regard to the Setting of Tolerances for Nutrient Values Declared on a Label. https://food.ec.europa.eu/safety/labelling-and-nutrition/food-information-consumers-legislation/guidance-documents_en.

[47] Heuer T., Auflage L. (2021). Fettzufuhr in Deutschland: Ergebnisse aus NVS II und NEMONIT. Zucker-Fette-Proteine: Makronährstoffe im Interdiszip linären Diskurs.

[48] Johner S., Thamm M., Schmitz R., Remer T. (2015). Current daily salt intake in Germany: Biomarker-based analysis of the representative DEGS study. Eur. J. Nutr..

[49] Federal Ministry of Food and Agriculture Nutri-Score. https://www.bmel.de/DE/themen/ernaehrung/lebensmittel-kennzeichnung/freiwillige-angaben-und-label/nutri-score/nutri-score_node.html.

[50] European Parliament, European Council Regulation (EC) No 1169/2011 of the European Parliament and of the Council of 25 October 2011 on the Provision of Food Information to Consumers, Amending Regulations (EC) No 1924/2006 and (EC) No 1925/2006 of the European Parliament and of the Council, and Repealing Commission Directive 87/250/EEC, Council Directive 90/496/EEC, Commission Directive 1999/10/EC, Directive 2000/13/EC of the European Parliament and of the Council, Commission Directives 2002/67/EC and 2008/5/EC and Commission Regulation (EC) No 608/2004. https://eur-lex.europa.eu/legal-content/EN/ALL/?uri=CELEX:32011R1169.

[51] Fardet A., Rock E. (2020). Ultra-processed foods and food system sustainability: What are the links?. Sustainability.

[52] Seferidi P., Scrinis G., Huybrechts I., Woods J., Vineis P., Millett C. (2020). The neglected environmental impacts of ultra-processed foods. Lancet Planet. Health.

[53] Andreani G., Sogari G., Marti A., Froldi F., Dagevos H., Martini D. (2023). Plant-based meat alternatives: Technological, nutritional, environmental, market, and social challenges and opportunities. Nutrients.

[54] Saerens W., Smetana S., Van Campenhout L., Lammers V., Heinz V. (2021). Life cycle assessment of burger patties produced with extruded meat substitutes. J. Clean. Prod..

